# Mesenchymal Stem/Stromal Cells Derived from Dental Tissues: A Comparative In Vitro Evaluation of Their Immunoregulatory Properties Against T cells

**DOI:** 10.3390/cells8121491

**Published:** 2019-11-22

**Authors:** María del Pilar De la Rosa-Ruiz, Marco Antonio Álvarez-Pérez, Víctor Adrián Cortés-Morales, Alberto Monroy-García, Héctor Mayani, Gladis Fragoso-González, Sara Caballero-Chacón, Daniel Diaz, Fernando Candanedo-González, Juan José Montesinos

**Affiliations:** 1 Mesenchymal Stem Cells Laboratory, Oncology Research Unit, Oncology Hospital, National Medical Center (IMSS), POST 06720 Mexico City, Mexico; tzotzilmvz@gmail.com (M.d.P.D.l.R.-R.); v.adrian.cortes@gmail.com (V.A.C.-M.); 2 Posgraduate in Production Sciences and Animal Health, National Autonomous University of Mexico (UNAM), 04510 Mexico City, Mexico; 3 Tissue Bioengineering Laboratory, Division of Graduate Studies and Research of the Faculty of Dentistry, National Autonomous University of Mexico (UNAM), 04510 Mexico City, Mexico; malvap6@gmail.com; 4 Immunology and Cancer Laboratory; Oncology Research Unit, Oncology Hospital, National Medical Center (IMSS), 06720 Mexico City, Mexico; albertomon@yahoo.com; 5 Hematopoietic Stem Cells Laboratory, Oncology Research Unit, Oncology Hospital, National Medical Center (IMSS), 06720 Mexico City, Mexico; hmayaniv@prodigy.net.mx; 6 Institute of Biomedical Research, Immunology Departament, National Autonomous University of Mexico (UNAM), 04510 Mexico City, Mexico; gladis@unam.mx; 7 Department of Physiology and Pharmacology, Faculty of Veterinary Medicine, National Autonomous University of Mexico (UNAM), 04510 Mexico City, Mexico; saracachas@hotmail.com; 8 Faculty of Veterinary Medicine, Autonomous University of Sinaloa, Culiacan Rosales, 80246 Sinaloa, Mexico; ddiaz@ciencias.unam.mx; 9 Department of Pathology, High Specialty Medical Unit (UMAE), National Medical Center (IMSS), 06720 Mexico City, Mexico; fa_candanedo@yahoo.com.mx

**Keywords:** gingival tissue, dental pulp, periodontal ligament, immunoregulation, mesenchymal stem/stromal cells, T cells

## Abstract

Bone marrow mesenchymal stem/stromal cells (BM-MSCs) have immunoregulatory properties and have been used as immune regulators for the treatment of graft-versus-host disease (GVHD). Human dental tissue mesenchymal stem cells (DT-MSCs) constitute an attractive alternative to BM-MSCs for potential clinical applications because of their accessibility and easy preparation. The aim of this in vitro study was to compare MSCs from dental pulp (DP-MSCs), gingival tissue (G-MSCs), and periodontal ligament (PDL-MSCs) in terms of their immunosuppressive properties against lymphoid cell populations enriched for CD3^+^ T cells to determine which MSCs would be the most appropriate for in vivo immunoregulatory applications. BM-MSCs were included as the gold standard. Our results demonstrated, in vitro, that MSCs from DP, G, and PDL showed immunoregulatory properties similar to those from BM, in terms of the cellular proliferation inhibition of both CD4^+^- and CD8^+^-activated T-cells. This reduced proliferation in cell co-cultures correlated with the production of interferon-γ and tumor necrosis factor alpha (TNF-α) and the upregulation of programmed death ligand 1 (PD-L1) in MSCs and cytotoxic T-cell-associated Ag-4 (CTLA-4) in T-cells and increased interleukin-10 and prostaglandin E_2_ production. Interestingly, we observed differences in the production of cytokines and surface and secreted molecules that may participate in T-cell immunosuppression in co-cultures in the presence of DT-MSCs compared with BM-MSCs. Importantly, MSCs from four sources favored the generation of T-cell subsets displaying the regulatory phenotypes CD4^+^CD25^+^Foxp3^+^ and CD4^+^CD25^+^CTLA-4^+^. Our results in vitro indicate that, in addition to BM-MSCs, MSCs from all of the dental sources analyzed in this study might be candidates for future therapeutic applications.

## 1. Introduction

Mesenchymal stem/stromal cells (MSCs) represent a heterogeneous population of progenitor cells capable of self-renewal and multipotentiality [1,2,3]. MSCs have immunoregulatory properties, making them special candidates for cell therapy, as they can reshape tissue under anti-inflammatory mechanisms through cell contact and growth factor and cytokine secretion [4,5]. In particular, MSCs have been shown to immunosuppress both CD4^+^ and CD8^+^ T-cells, which is important because they are effector cells in immunological diseases such as graft-versus-host disease (GVHD) [6]; therefore, MSCs have been proposed as potential cells for clinical application.

Bone marrow (BM) is the most studied source of MSCs [7], and BM-MSCs have been used in clinical cell therapy protocols to reduce signs of GVHD [6,7,8]. However, BM has some disadvantages, such as the process of finding donors and obtaining samples involving an invasive, painful, and expensive procedure [9]. The umbilical cord, as a discarded tissue at birth, represents an appropriate source of MSCs because its phenotype is similar to other sources, as well as because of the relatively high cell yields [10]. Similarly, adipose tissue is a stable source of MSCs as a result of its less invasive procedures, high yield, and lack of ethical issues [11]. Taking advantage of these benefits, it is important to mention that both sources have been applied in clinical protocols [10,12]. Our research team reported that umbilical cord blood (UCB) MSCs are an alternative to BM-MSCs because both have a similar capacity for T-cell immunosuppression; however, in that same study, UCB-MSCs were obtained at a low rate [13]; therefore, despite being an accessible source, a large number of samples would have to be processed to obtain a usable amount of MSCs. It is important to isolate MSCs from equally accessible sources but to obtain the MCSs at a high rate. In adults, MSCs have been found in dental tissues (DT-MSCs), such as dental pulp (DP-MSCs) [3], gingival tissue (G-MSCs) [14,15], and periodontal ligament (PDL-MSCs) [16]; it has been established that these sources are easily accessible and that MSCs can be obtained through minimally invasive isolation techniques that do not put the donor at risk, can be isolated in an autologous manner, and can be obtained at a high rate [15,16,17]. However, to date, we do not know if DT-MSCs have the same immunosuppressive potential as BM-MSCs; it is even more important to determine which MSCs provide the best immunosuppressive cell therapy.

Previous in vitro studies have established that direct contact between MSCs and CD3^+^ T-cells is necessary to inhibit the proliferation of CD4^+^ and CD8^+^ lymphocytes [18]. In this regard, the expression of some immunosuppressive molecules in MSCs, such as the programmed death ligand (PD-L1) and the human leukocyte antigen G-1 (HLA-G1), has been demonstrated [19,20]. However, it has also been shown MSCs can secrete immunosuppressive molecules such as interleukins 4 and 10 (IL-4 and IL-10), indoleamine 2,3-dioxygenase (IDO), and prostaglandin E_2_ (PGE_2_) [5,21,22], which participate in immunosuppressive mechanisms.

It has also been reported that MSCs promote the in vitro generation of T regulatory cells (Tregs) with the CD4^+^CD25^+^ forkhead box P3 (Foxp3^+^) or CD4^+^CD25^+^CTLA-4^+^ phenotype [23,24]; on the contrary, it has also been shown that BM-MSCs do not induce the expression of the transcription factor Foxp3^+^ [25]. These contradictory findings could derive from the T-cell population used and the absence or presence of cell contact with BM-MSCs [26]. In this way, MSCs exert their immunoregulatory properties under a proinflammatory stimulus with cytokines, such as TNFα and interferon (IFN)γ [4,27], thus decreasing T-cell proliferation and inducing Treg differentiation, which is related with the expression of surface molecules and soluble mediators by MSCs.

Considering the above, the present in vitro study aims to contribute to the knowledge of the immunosuppressive properties of BM-, DP-, G-, and PDL-MSCs on CD3^+^ T-cells; for this purpose, we conducted a comparative study of MSCs derived from the four cell sources in relation to their potential to inhibit the proliferation of CD3^+^ T-cells and the expression of both surface and secreted immunosuppressive molecules. We also analyzed the capacity of MSCs to generate Tregs in co-culture. To our knowledge, this is the first in vitro study comparing the immunosuppressive properties of MSCs derived from these four cell sources to determine which one could be the most appropriate for immunoregulatory therapy.

## 2. Materials and Methods

### 2.1. Obtaining Biological Samples

BM-MSC samples (n = 5) were obtained from hematologically healthy adult volunteer donors according to the guidelines of the Traumatology and Orthopedics Hospital of the Mexican Social Security Institute (IMSS—its acronym in Spanish) registered under protocol 1411. Samples of DP-MSCs (n = 5), G-MSCs (n = 5), and PDL-MSCs (n = 5) were obtained by an explant tissue culture system from adult patients who underwent third molar extraction at the Maxillofacial Surgery Clinic of the Division of Postgraduate Studies and Research (DEPeI—its acronym in Spanish) at the School of Dentistry of the National Autonomous University of Mexico. Patients were informed about the study prior to obtaining their consent and donation of extracted dental organs, and the protocol was approved by the Research and Ethics Committee of the School of Dentistry (CIE/1110/2017). CD3^+^ T-cells were obtained from peripheral blood mononuclear cells (PBMCs) from healthy adult donors.

### 2.2. Isolation and Culture of BM-MSCs, DP-MSCs, G-MSCs, and PDL-MSCs

#### 2.2.1. Isolation and Culture of BM-MSCs

Samples were obtained from BM aspirates from the iliac crest and placed in a 50 mL tube (Corning, New York, NY USA) with 15 mL of Roswell Park Memorial Institute (RPMI)-1640 culture medium (ThermoFisher, Waltham, MA USA) containing 10% fetal bovine serum (FBS) and then washed with phosphate-buffered solution (PBS, ThermoFisher, Waltham, MA, USA). A density gradient was prepared with Ficoll-Paque Plus, density 1.077 + 0.001 g/mL (GE Healthcare Bio-Sciences AB, Uppsala, Sweden). The cells were centrifuged at 300× *g* for 30 min, and the interface was washed with PBS containing 3% FBS and 1 mM EDTA. The mononuclear cell (MNC) pellet was resuspended in low-glucose Dulbecco’s Modified Eagle’s Medium (lg-DMEM) supplemented with 15% FBS. The total number of nucleated cells and their viability were determined by counting with Turck’s solution and trypan blue (ThermoFisher), respectively. From 5 to 10 × 10^6^ MNCs were seeded in a 100 mm Petri dish (Corning) and incubated at 37 °C with 5% CO_2._ After four days, a PBS wash was performed to remove non-adherent cells, changing the medium twice per week. When the cultures reached 80%–90% confluence, the cells were harvested for reseeding and cryopreservation. The MSCs of passages 3 and 4 were used for the experiments.

#### 2.1.2. Isolation and Culture of MSCs from a Dental Tissue Explant Tissue Culture System

After the third molar exodontia, the periodontal ligament covering the roots of the dental organ and the gingival tissue (oral mucosa) were dissected, which was firmly adhered to the periosteum; lastly, the tooth was sectioned with a diamond disk to expose the pulp cavity and thus extract the dental pulp. The three tissues were separately mechanically disintegrated and placed in a six-well plate (Corning), embedded in 1 mL of alpha-Dulbecco’s Modified Eagle’s Medium (αMEM) supplemented with 10% FBS, 2 mM L-glutamine, 100 IU/mL penicillin, 100 µg/mL streptomycin, and 100 μg/mL gentamicin (GIBCO BRL, Carlsbad, CA, USA), where they were kept for 2 to 5 weeks, replacing the culture medium every third day. Upon reaching a confluence of 80%, the cells were harvested by incubating them in trypsin-0.02% EDTA (GIBCO, BRL) at 37 °C with 5% CO_2_ for 5 min; later, MSCs from each tissue were counted in a Neubauer chamber (Sigma-Aldrich, St. Louis, MI, USA) with viability staining (trypan blue). Lastly, 1 × 10^6^ MSCs from each tissue were frozen-embedded in freezing medium containing 10% dimethylsulfoxide (Sigma-Aldrich) and cryopreserved in 2 mL microtubes (Corning) in liquid nitrogen for later use. The MSCs of passages 3 and 4 were used for the experiments.

### 2.3. Characterization of Mesenchymal Stem Cells

#### 2.3.1. Immunophenotype

The immunophenotypic characterization of BM-MSCs and DT-MSCs was performed according to previously described protocols. Monoclonal antibodies conjugated to FITC, PE, or APC against CD73, CD90, and CD45 (BD Biosciences, San Diego, CA, USA), CD105, CD13, and CD14 (Buckingham, UK), and human leukocyte antigen (HLA)-ABC, HLA-DR, CD31, and CD34 (Invitrogen, Carlsbad, CA, USA) were used as described in the Flow Cytometry Analysis section.

#### 2.3.2. Morphological Analysis

To identify morphological differences between BM-MSCs and DT-MSCs, 0.3 × 10^5^ cells/cm^2^ were reseeded in P-35 boxes (Corning); upon reaching 40% confluence, the cells were stained with toluidine blue (Sigma-Aldrich) and evaluated using phase-contrast microscopy (n = 5).

#### 2.3.3. Differentiation Capacity: Adipogenic

For adipogenic differentiation, 0.8 × 10^5^ cells suspended in low-glucose Dulbecco’s Modified Eagle’s Medium (ThermoFisher-Gibco) containing 10% FBS were seeded in 35 mm Petri dishes (Corning). When 60% confluence was reached, the cells were induced with MesenCult Adipogenic Differentiation Kit medium (StemCells Technologies, Vancouver, Canada) and incubated for 21 days, changing the medium twice per week. To visualize adipocytes and lipid vacuoles, cytochemical staining was performed with Oil Red O (Sigma-Aldrich).

#### 2.3.4. Osteogenic

For osteogenic differentiation, 0.8 × 10^5^ cells suspended in lg-DMEM (ThermoFisher-Gibco) supplemented with 10% FBS were seeded in 35 mm Petri dishes (Corning). When 60% confluence was reached, induction was initiated with StemPro osteogenic medium (Gibco, Carlsbad, California, CA, USA), and the cells were incubated for 21 days, changing the medium twice per week. Finally, alkaline phosphatase activity was detected using SIGMA FAST^™^ BCIP/NBT (5-bromo-4-chloro-3-indolyl phosphate/nitro blue tetrazolium) (Sigma-Aldrich).

#### 2.3.5. Chondrogenic

For osteogenic differentiation, 3 × 10^5^ cells suspended in lg-DMEM (ThermoFisher-Gibco) at 10% FBS were placed in 15 mL tubes and centrifuged at 900 rpm to obtain a cell pellet to which chondrogenic medium (Cambrex Bio Science, Walkersville, Inc., Walkersville, MD, USA) supplemented with 10 ng/mL transforming growth factor beta (TGF-β) (Cambrex Bio Science, Walkersville, MD, USA) was added. The cell pellet was incubated for 28 days, changing the medium twice per week. The resulting cell micromasses were fixed, dehydrated, and embedded in paraffin, after which 4 μm thick sections were obtained with a microtome. Subsequently, cytochemical staining was performed with Alcian Blue (Sigma-Aldrich).

### 2.4. Obtaining CD3^+^ T-cells

To isolate CD3^+^ cells, PBMCs were obtained from healthy donors using a density gradient (Ficoll-Paque Plus, density 1.077 + 0.001 g/mL; GE Healthcare Bio-Sciences AB, Uppsala, Sweden); CD3^+^ cells were isolated from this population by positive magnetic selection using CD3 MicroBeads and MACS MS columns (Miltenyi Biotec, Bergisch Gladbach, Germany) according to the Supplementary Materials. The purity of the CD3^+^ population was 97%, as determined by flow cytometry. CD3^+^ cells were incubated in RPMI-1640 medium supplemented with 10% FBS, 2 mM L-glutamine, 100 IU/mL penicillin, 100 μg/mL streptomycin, and 100 μg/mL gentamicin (GIBCO BRL) for 24 h.

### 2.5. Activation of CD3^+^ T-cells

CD3^+^ T-cells were activated in co-culture medium 50% lg-DMEM/50% RPMI supplemented with 10% FBS, 2 mM L-glutamine, 100 IU/mL penicillin, 100 μg/mL streptomycin, and 100 μg/mL gentamicin (GIBCO BRL) with Dynabeads anti-CD3/CD28 T-cell expander (Invitrogen, California, CA, USA) at a 1:1 ratio (25 μL of Dynabeads/1 × 10^6^ CD3^+^ T-cells), which was added to the monolayer of MSCs from different sources (this technique is described Section 2.6.). The cultures were maintained and evaluated after four days by flow cytometry. For the proliferation assay, activated CD3^+^ cells were stained with 5(6)-carboxyfluorescein succinimidyl ester (CFSE, Thermo Fisher Scientific).

### 2.6. Co-culture of MSCs/CD3+ T-cells

The co-culture system consisted of adding αCD3/CD28-activated T-cells to a single layer of MSCs at a ratio of one MSC to one T-cell. Cultures in 24-well plates (Corning) with activated CD3+ T-cells were used as the control. Then, 1.5 × 10^5^ activated CD3+ T-cells were added to a monolayer of 1.5 × 105 BM-MSCs, DP-MSCs, G-MSCs, or PDL-MSCs; antibody-coupled beads were present during the entire culture time. In the proliferation and cytokine and surface molecule expression assays, CD3+ T-cells activated in the absence of MSCs were used as a positive control. MSCs cultured in the absence of CD3+ T-cells were used as the basal expression control for surface and secreted molecules. Cultures were maintained and evaluated after four days by flow cytometry.

### 2.7. Proliferation Assay

After four days of co-culture, the cells were harvested and flow cytometry was performed, which consisted of washing the cells with physiological saline solution (PSS), blocking the cells with Fc Blocking Reagent Human (Miltenyi Biotec, Bergisch Gladbach, Germany), resuspending the cells in 100 μL of PBS containing 1 mM EDTA and 3% FBS and incubating the cells at 4 °C with antibodies (CD3, CD4, and CD8 conjugated to PE (BD Biosciences, Franklin Lakes, NJ, USA) for 20 min; then, the cells were washed with 1 mL of cytometry buffer (PBS containing 1 mM EDTA and 3% FBS). The cells were analyzed in a FACS Canto II Flow Cytometer (BD Biosciences), where 10,000 events were captured per sample. The data were analyzed with FlowJo 10 software (Ashland, OR, USA), and the average fluorescence intensities were normalized using 1 as the control for each experiment. CD3^+^ T-cells activated in the absence of MSCs were used as the positive control and were considered as 100% proliferation. The proliferation levels observed in co-cultures were normalized to this control. FlowJo 10 software was used for the analysis.

### 2.8. Expression of Immunoregulatory Molecules

After four days of co-culture, the cells were harvested and the flow cytometry method described above was performed; in this case, the cells were fixed with FACS Lysing Solution (BD Biosciences) to evaluate the expression of CD4^+^, CD25^+^, and CTLA-4^+^ surface molecules on CD3^+^ T-cells with monoclonal antibodies conjugated to FITC, PE, or APC (BD Biosciences) and intracellular Foxp3 expression in CD3^+^ T-cells with an antibody conjugated to APC (eBioscience, San Diego, CA, USA). The expression of PD-L1 (antibody conjugated to PE; BD Biosciences) on MSCs labeled with CD90 antibodies conjugated to APC was also determined. The cells were analyzed in a FACS Canto II Flow Cytometer (BD Biosciences), where 10,000 events were captured per sample. The data were analyzed with FlowJo 10 software, and the average fluorescence intensities were normalized using one as the control for each experiment.

### 2.9. Quantitative Analysis of Soluble Molecules

#### 2.9.1. Cytokines

To determine cytokine levels, a cytometric bead array (CBA) was used. This technique uses beads conjugated to specific antibodies against various molecules, allowing the simultaneous determination of seven cytokines in a single sample of cell co-culture supernatant. Its main value is the ability to combine the fast and simultaneous reading of several complex parameters in an objective and accurate manner. To determine the concentration of secreted cytokines, supernatants were obtained four days after co-culture using the various conditions, including non-activated CD3^+^ T-cells. MSCs in the absence of CD3^+^ were used as the negative control, and CD3^+^ activated T-cells in the absence of MSCs were used as the positive control. Supernatants were stored at − 70 °C until use. Cytokine analysis was performed using a Cytometric Bead Array kit (BD Biosciences) according to the supplier’s instructions. Thus, 10 μL of capture beads that recognized each cytokine (TNFα, IFNγ, IL-10, and IL-4) was mixed; this mixture was incubated at room temperature and protected from light for 30 min. A standard curve was developed by preparing serial dilutions of each secreted molecule (0–500 pg/mL). Later, 50 μL of each supernatant or point of the standard curve was mixed with 50 μL of capture beads; this mixture was incubated at room temperature and protected from light for 3 h. Then, a wash was performed with 1 mL of wash buffer, and the pellet was resuspended in 300 μL of the same buffer. The samples were analyzed on a FACS Canto II Flow Cytometer (BD Biosciences) and analyzed with LegendPlex v7.1 software (San Diego, CA, USA).

#### 2.9.2. Prostaglandin E_2_

The concentration of PGE_2_ was determined by enzyme-linked immunosorbent assay with an ELISA kit for human PGE_2_ (Invitrogen) according to the supplier’s instructions. The supernatant of MSCs cultured in the absence of activated CD3⁺ T-cells was used as the control. A standard PGE_2_ curve was prepared using serial dilutions of 0.3 to 4.0 ng/mL, subsequently adding 100 μL of the samples or of each point of the curve to microplate wells; each sample was measured in duplicate. To each well, previously coated with goat anti-mouse polyclonal antibody, 50 μL of alkaline phosphatase-labeled PGE_2_ and 50 μL of mouse monoclonal anti-PGE_2_ antibody were added, except for the blank wells. The plate was incubated at room temperature with stirring for 2 h; then, the wells were washed to remove non-adhered cells. The alkaline phosphatase substrate (para-nitrophenyl phosphate, pNPP) was then added, and the plate was incubated for 1 h. The plate was read on an ELISA plate reader (DYNATEC MR5000) at 412 nm.

### 2.10. Statistical Analysis

For the statistical analysis, one-way ANOVA was used, followed by Tukey’s multiple comparisons test to determine significant differences in the proliferation response (CD3, CD4, and CD8) and changes in the expression of CD4^+^CTLA-4^+^, Tregs (CD4^+^, CD25^+^, Foxp3^+^, and CD4^+^, CD25^+^, CTLA-4^+^) and in the production of interleukins and factors in different cultures with MSCs from different sources. To determine if the co-culture conditions caused changes in PD-L1^+^ and PGE_2_ expression compared with the control group, we used a two-way ANOVA followed by Tukey’s multiple comparisons test with statistical hypothesis testing. All analyses and graphs were performed in Prism 8 (GraphPad Software, Inc., San Diego, CA, USA). A *p*-value < 0.05 was considered significant.

## 3. Results

### 3.1. MSCs from Dental Tissues Show Immunophenotypes and Differentiation Capacities Similar to Those from Bone Marrow

**Figure 1:**
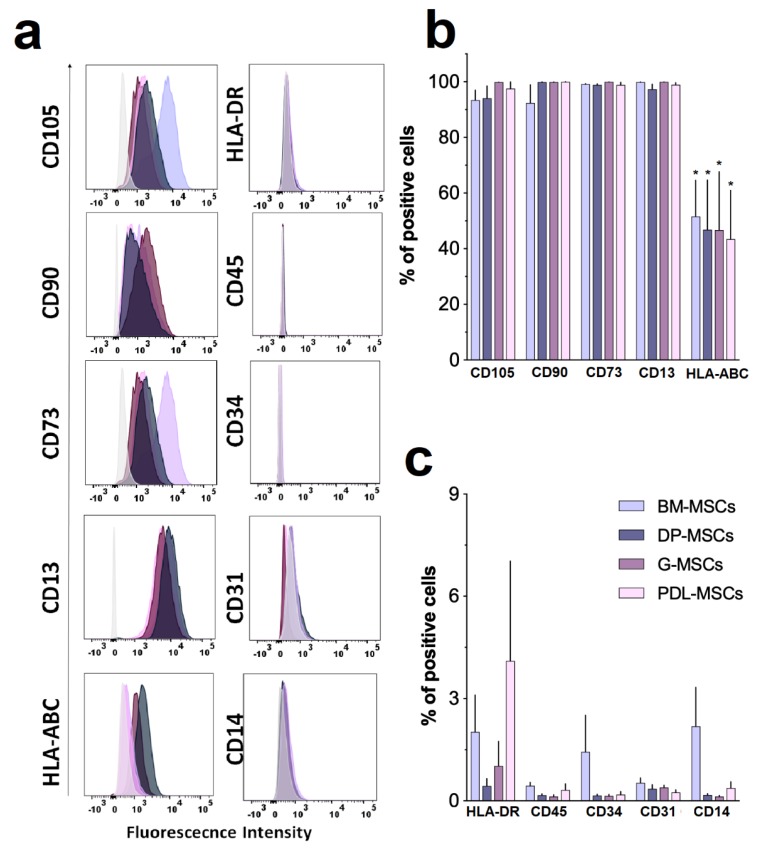
The expression of cell markers in dental tissue–mesenchymal stem cells (DT-MSCs) was determined by flow cytometry. (**a**) Representative histograms for one experiment (gray histogram: isotype control, (**b**) DT-MSC cultures show a high expression of surface markers characteristic of MSCs (CD105, CD73, CD90, and CD13), moderate expression of human leukocyte antigen (HLA)-ABC, and (**c**) negative expression for hematopoietic markers (CD45, CD34, and CD14), endothelial markers (CD31) and HLA-DR; bone marrow (BM)-MSCs (n = 5), dental pulp (DP)-MSCs (n = 5), gingival tissue (G)-MSCs (n = 5), and periodontal ligament (PDL)-MSCs (n = 5). The results represent the mean ± SD and correspond to the percentage (%) of cells positive for each particular antigen. *Significant difference (*p <* 0.05) compared with CD105, CD73, CD90, and CD13.

**Figure 2:**
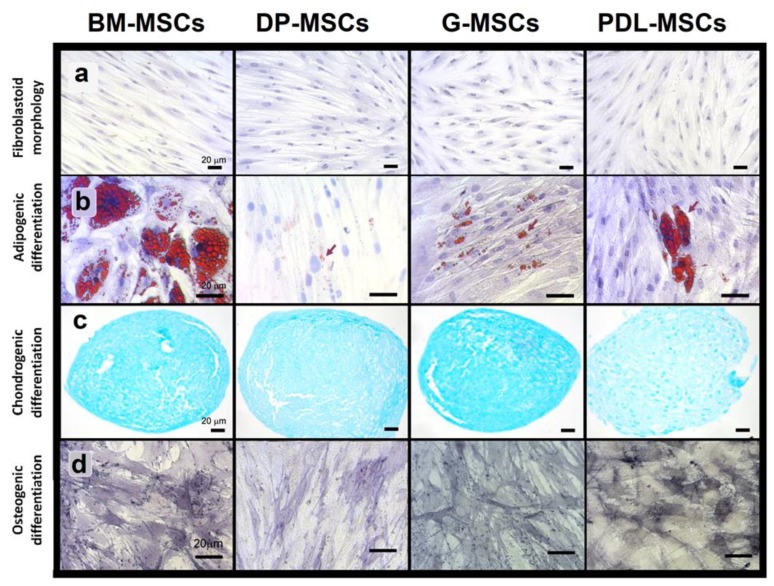
Morphology and differentiation capacity of mesenchymal stromal cells (MSCs) from bone marrow (BM), dental pulp (DP), gingival tissue (G), and periodontal ligament (PDL). Appearance of adherent cells with fibroblastoid morphology observed in cultures from the indicated sources (toluidine blue staining; magnification: 20×) (**a**). MSCs from the four cell sources (BM, n = 5; DP, n = 5; G, n = 5; and PDL, n = 5) were cultured in adipogenic, chondrogenic and osteogenic induction media for 35, 28, and 21 days, respectively (**b–d**). Adipogenic differentiation was indicated by the accumulation of neutral lipid vacuoles (Oil Red O; magnification: 40×) (**b**). Chondrogenic differentiation was indicated by chondrogenic matrix stained with alcian blue (cryosections from pelleted micromass; magnification: 20X) (**c**). Osteogenic differentiation was indicated by positive alkaline phosphatase enzyme staining (magnification: 40×) (**d**). One representative experiment is shown.

Individual experiments were performed with BM-MSCs (n = 5), DP-MSCs (n = 5), G-MSCs (n = 5) and PDL-MSCs (n = 5) to determine their immunophenotype, morphology, and differentiation capacity—adipogenic, osteogenic, and chondrogenic (Figure 1 and Figure 2). Similarly to BM-MSCs, DT-MSCs expressed high levels of surface markers characteristic of MSCs—CD105, CD90, and CD73—as established by the International Society for Cellular Therapy (ISCT) [7]. Interestingly, high CD13 expression was observed, as we have previously reported for MSCs derived from other sources [13,28]. Additionally, DT-MSCs expressed moderate levels of HLA-ABC, were negative for HLA-DR expression and did not express hematopoietic markers, such as CD34, CD45, and CD14 or endothelial markers such as CD31 (Figure 1). In turn, all MSCs derived from the four cell sources showed fibroblastoid morphology and differentiation capacity toward adipogenic, chondrogenic, and osteogenic lineages. However, in DP-MSCs, we did not observe cells with adipocytic morphology (Figure 2b); only fibroblastic cells with Oil Red O-positive foci in the cytoplasm were detected.

### 3.2. CD4+ and CD8+ T-cell Proliferation Was Similarly Inhibited by Co-culture with MSCs from BM and Dental Tissues

**Figure 3:**
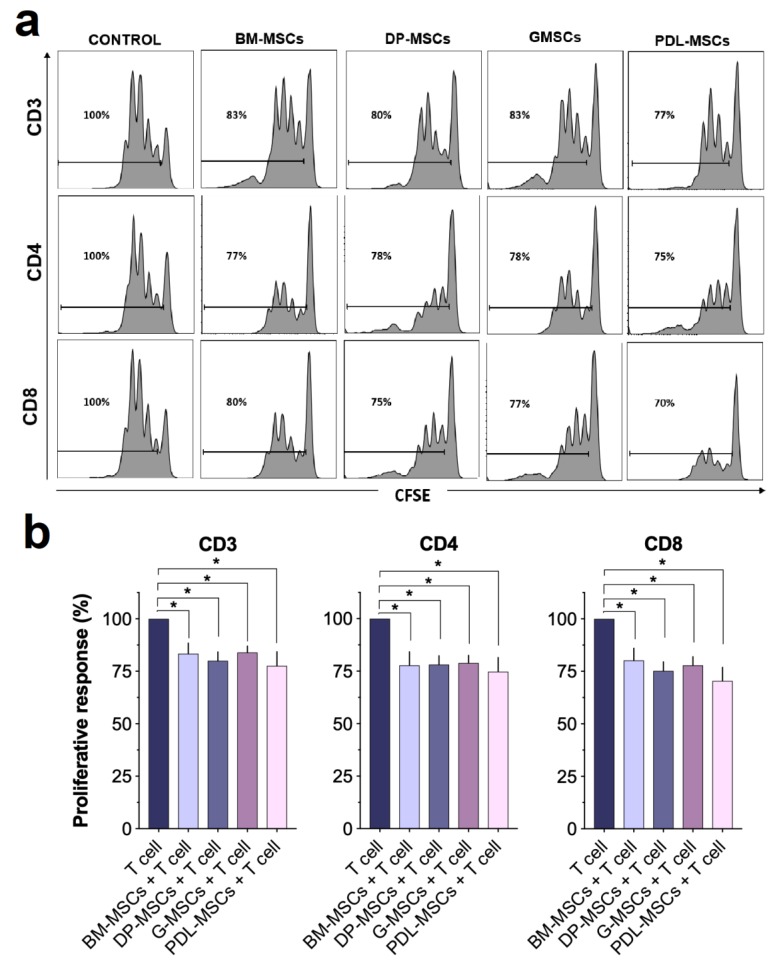
DT-MSCs and BM-MSCs similarly decrease T-cell proliferation. CD3^+^ cells were activated with anti-CD3/CD28 and cocultured in the presence of BM-MSCs, DP-MSCs, G-MSCs, or PDL-MSCs at a 1:1 ratio of MSCs: CD3^+^ cells. The proliferation rate of activated CD3^+^ cells in the absence of MSCs was used as the positive control (100% proliferation; n = 5). The rate of incorporation of CFSE (carboxyfluorescein succinimidyl ester) was determined in CD3^+^, CD4^+^, and CD8^+^ populations after four days of culture. (**a**) Representative histograms for one experiment. (**b**) Data are shown as the mean ± SEM of the proliferation rate (n = 5, individual experiments). *Significant difference, *p* < 0.05.

Previous studies in our laboratory demonstrated the importance of evaluating the immunosuppressive capacity of BM-MSCs on CD3^+^ T-cell-enriched populations in co-cultures with cell contact between the two populations [13,18]. Therefore, we analyzed the effect of DT-MSCs on CD3^+^ T-cell-enriched populations using co-cultures allowing cell contact. For this, CD3^+^ cells (≥97% purity) were isolated and activated with anti-CD3/CD28 in the presence or absence of BM-MSCs, DP-MSCs, G-MSCs, or PDL-MSCs (Figure 3). T-cells that were activated in the absence of MSCs were used as positive controls, set at 100% proliferation. The levels of proliferation observed in the co-cultures were normalized to this control. As seen in Figure 3a,b, BM-MSCs, DP-MSCs, G-MSCs, and PDL-MSCs reduced the proliferation of CD3^+^ T-cells (83.39% ± 12.54%, 80.05 ± 10.42%, 83.93 ± 7.74%, and 77.5% ± 16.91, respectively; *p* < 0.05), CD4^+^ T-cells (77.75% ± 16.17%, 78.11% ± 10.52%, 78.88% ± 9.25%, and 74.79% ± 16.76%, respectively; *p* < 0.05) and CD8^+^ T-cells (80.25% ± 14.46%, 75.25% ± 10.63, 77.84% ± 10.23%, and 70.50% ± 16.02%, respectively; *p* < 0.05). These results suggest that BM-MSCs, DP-MSCs, G-MSCs, and PDL-MSCs have a similar ability to inhibit T-cell proliferation.

### 3.3. Increased Expression of the Immunosuppressive Molecules CTLA-4 and PD-L1

**Figure 4:**
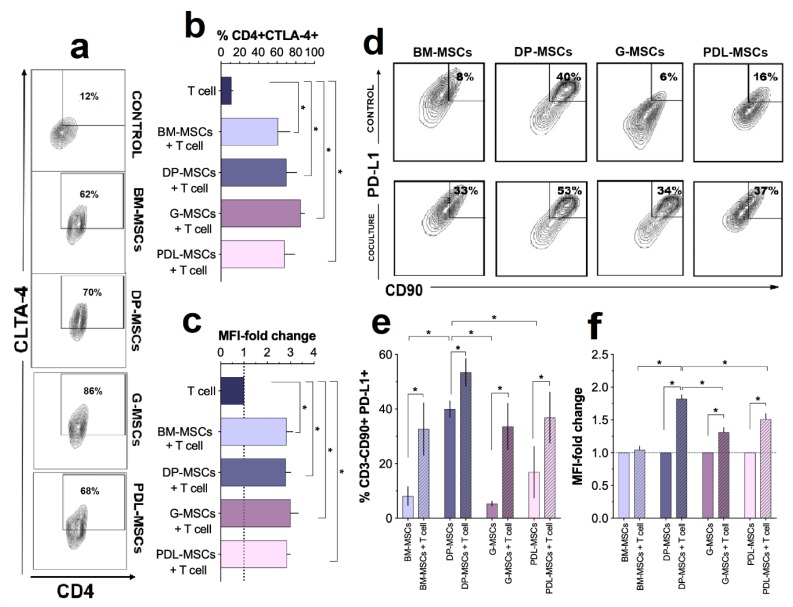
Increased expression of cytotoxic T-cell-associated Ag-4 (CTLA-4) in CD4^+^ T-cells and programmed death ligand 1 (PD-L1) in MSCs. CD3^+^ T-cells were activated with anti-CD3/CD28 and cocultured in the presence of BM-MSCs, DP-MSCs, G-MSCs or PDL-MSCs at a 1:1 ratio of MSCs:T-cells. The expression of CTLA-4 in CD3^+^CD4^+^ cells cultured in the absence of MSCs was considered as the basal expression (Control). The percentage of CD3^+^CD4^+^CTLA-4^+^ cells and the fold increase in the CTLA-4 mean fluorescence intensity (MFI) were determined in the CD4^+^ population after four days of culture (n = 5). (**a**) Dot plots are representative of one experiment with the CD3^+^CD4^+^CTLA-4^+^ population. (**b**) Data are shown as the mean ± SEM of the percentage of CD4^+^ CTLA-4^+^ cells. (**c**) Fold increase in the CD4^+^ MFI of the CD4^+^CTLA-4^+^ population. PD-L1 expression in MSCs cultured in the absence of activated CD3^+^ T-cells was considered as the basal expression (Control). The percentage and MFI of PD-L1^+^ cells were determined in the CD3^-^CD90^+^ population after four days of culture (n = 5). (**d**) Dot plots are representative of one experiment. (**e**) Data are shown as the mean ± SEM of the percentage of CD3^-^CD90^+^PD-L1^+^ cells. (**f**) Fold increase in the PD-L1 MFI in the CD3^-^CD90^+^PD-L1^+^ population. * Significant difference at *p* < 0.05.

We previously demonstrated in a co-culture system that BM-MSCs increase the expression of CTLA-4 on CD4^+^ T-cells, favoring their immunosuppression [18]. With this background, we analyzed the expression of CTLA-4 on CD4^+^ T-cells after four days of co-culture with DT-MSCs to determine if this molecule participates in the decreased proliferation observed in T-cells. Co-cultures were performed with BM-MSCs as controls, measuring the rate and increase in mean fluorescence intensity (MFI) for CTLA-4⁺ T-cells in the CD4⁺ population (Figure 4a–c). The expression of CTLA-4 in activated T-cells cultured in the absence of MSCs was considered as 100%.

Co-cultures of BM-MSCs, DP-MSCs, G-MSCs, and PDL-MSCs showed a significant increase in the percentage of CD4^+^CTLA-4^+^ cells (61% ± 22, 70% ± 9.73, 85% ± 8.04, and 68% ± 7.12, respectively) compared with the control (11% ± 2.63; *p <* 0.05.) (Figure 4a,b). Notably, there were no significant differences between the increases observed for MSCs from the different sources (Figure 4b). Similarly, we observed a significant increase in the MFI of CTLA-4 in CD4^+^CTLA-4^+^ MSCs from BM, PD, G, and PDL (2.93-, 2.80-, 3.02-, and 2.85-fold, respectively; *p* < 0.05), without differences between MSCs from different sources (Figure 4c). These results suggest that the presence of DT-MSCs, similarly to BM-MSCs, promotes an increase in the expression of CTLA-4 in T-cells and that this molecule can contribute to reducing the proliferation of these T-cells in co-cultures.

We also previously determined that PD-L1 is expressed in BM-MSCs and that, through cell–cell contact, it participates in the decrease in T-cell proliferation [18]. Due to this, we evaluated the expression of PD-L1 in DP-MSCs, G-MSCs, and PDL-MSCs and compared its expression with that in BM-MSCs in co-cultures with activated T-cells (Figure 4d–f). The percentage and MFI of PD-L1^+^ in MSCs cultured in the absence of T-cells were considered as basal expression. The results showed a significant increase (*p <* 0.05) in the basal expression of PD-L1^+^ in DP-MSCs (CD3^-^CD90^+^PD-L1^+^; 40% ± 5.3) compared with that in MSCs from the other sources (BM: 8.1% ± 6.02; G: 5.4% ± 1.5; PDL: 16.9% ± 16.3). The presence of activated T-cells in co-cultures significantly increased (*p* < 0.05) the percentage of PD-L1^+^ in MSCs from all sources: BM (32.7% ± 14.9), DP (53.5% ± 8.7), G (33.6% ± 14.6), and PDL (36.9% ± 16.13) (Figure 4d,e). Although a trend toward a higher percentage was observed for the expression of PD-L1 in DP-MSCs, there were no significant differences when compared with PD-L1 expression in MSCs from the other sources. Regarding the increase in PD-L1 MFI in the CD3^-^CD90^+^PD-L1^+^ population, in co-cultures, significant increases were observed (*p* < 0.05) in those with MSCs isolated from DP (1.82-fold), G (1.32-fold), and PDL (1.50-fold) but not from BM (1.03-fold; Figure 4f). Interestingly, the difference in the increase in the expression of PD-L1 in DP-MSCs was significant (*p* < 0.05) when compared with that in MSCs from the other sources.

These results show greater PD-L1 expression in DP-MSCs than in those from BM, G and PDL, which could suggest differences in the immunosuppressive mechanisms of T-cells by DP-MSCs compared with MSCs from the other sources.

### 3.4. Cytokine and PGE_2_ Secretion in MSC Co-cultures

**Figure 5:**
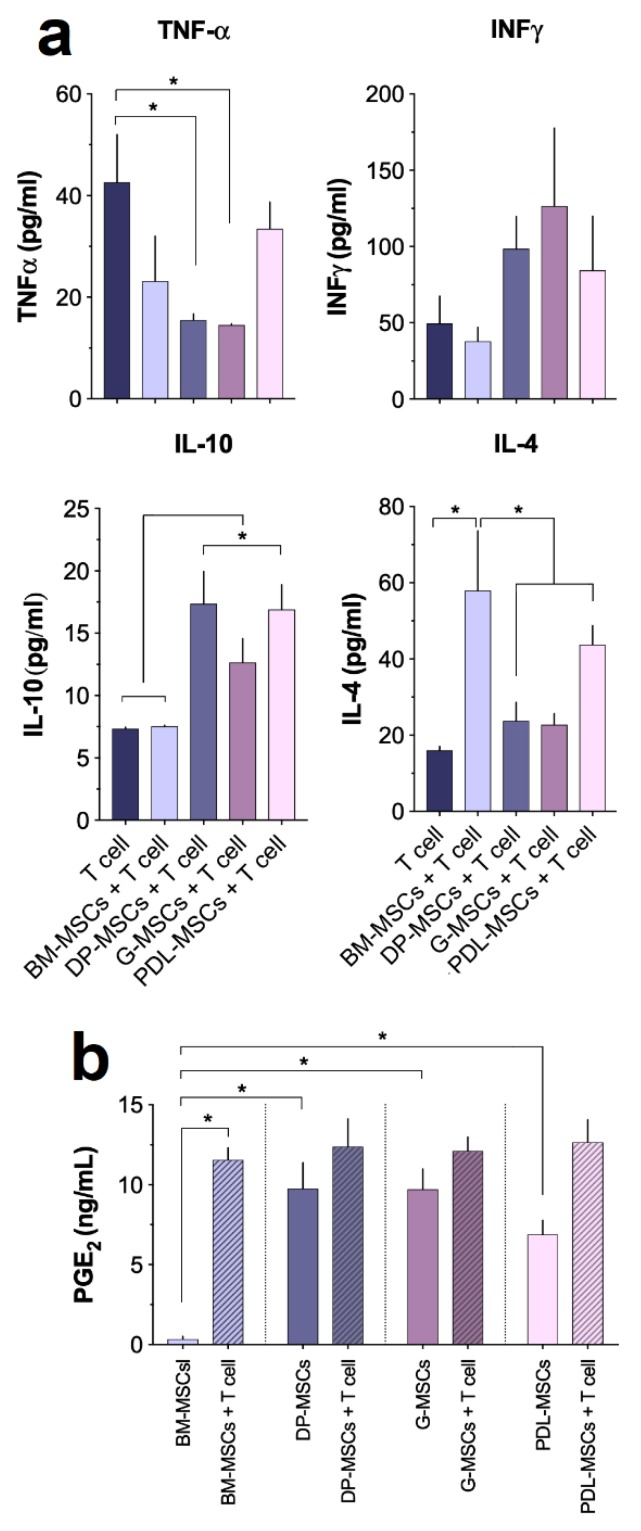
Molecule expression is differentially induced in co-cultures with MSCs from BM and dental tissues. CD3^+^ T-cells activated with anti-CD3/CD28 were cultured in the presence or absence of BM-MSCs or DP-MSCs or G-MSCs or PDL-MSCs at a 1:1 ratio of MSCs:T-cells. After four days of co-culture, TNFα, IFNγ, IL-10, and IL-4 were determined in the conditioned medium by cytometric bead array and PGE_2_ by ELISA. (**a**) The concentration of cytokines detected in the conditioned medium of CD3^+^ T-cells in the absence of MSCs was considered as basal expression for tumor necrosis factor alpha (TNFα), interferon (IFN)γ, interleukin (IL)-10 and IL-4, (**b**) while for PGE_2_, these were the concentrations detected in the conditioned medium of MSCs cultured in the absence of CD3^+^ T-cells. The data are presented as the mean ± SEM of the concentrations of the analyzed molecules (n = 5, individual experiments). * Significant difference at *p* < 0.05. PGE_2,_ prostaglandin E_2_; ELISA, enzyme-linked immunosorbent assay.

Previous studies have confirmed the importance of IFNγ and TNFα [4] in inducing BM-MSCs’ immunosuppressive properties. In turn, the decreased proliferation we observed could be related to the presence of Th2-type immunosuppressive cytokines such as IL-4 and IL-10 [4]. Based on the above, we evaluated the presence of cytokines that could be involved in the activation of the immunosuppressive response of MSCs (IFNγ and TNFα) and in the observed decrease in T-cell proliferation (IL-4 and IL-10). The concentration of cytokines detected in the conditioned medium of activated T-cells cultured in the absence of MSCs was considered the control. Concentrations of TNFα and IFNγ were detected in all conditioned media of co-cultures with MSCs (Figure 5a); however, only co-cultures with DP-MSCs and G-MSC showed significant decreases in TNFα (15.53 pg/mL ± 1.88 and 14.55 pg/mL ± 0.35, respectively; *p* < 0.05) compared with the control (42.60 pg/mL ± 14.56). Regarding IFNγ, there was an increasing trend in the concentration of this cytokine in dental tissue cultures, but it was not statistically significant (Figure 5a). In contrast, higher concentrations of IL-10 (*p* < 0.05) were detected in conditioned media from co-cultures with DP-MSCs (17.37 pg/mL ± 3.99), G-MSCs (12.65 pg/mL ± 2.99), and PDL-MSCs (16.09 pg/mL ± 3.07) compared with those with BM-MSCs (7.54 pg/mL ± 0.12) and the control (7.35 pg/mL ± 0.18) (Figure 5a). Interestingly—and contrary to what was observed for IL-10—a higher concentration of IL-4 (*p* < 0.05) was detected in co-cultures with BM-MSCs (57.95 pg/mL ± 24.26) than in those from DP-MSCs (23.74 pg/mL ± 7.57), G-MSC (22.75 pg/mL ± 4.44), and PDL-MSCs (43.75 pg/mL ± 7) (Figure 5a). These results suggest that the presence of TNFα and IFNγ differentially induce the secretion of immunosuppressive cytokines in DT-MSCs compared with BM-MSCs, with IL-4 being preferentially produced in co-cultures in the presence of the latter, while IL-10 is preferentially produced in those with DT-MSCs, which could be related to mechanisms other than cytokine-mediated immunosuppression between the two types of MSCs.

Similarly, PGE_2_ is a molecule that decreases T-cell proliferation, stimulates the secretion of IL-4 and IL-10 and promotes T-cell differentiation toward lymphocytes with the regulatory phenotype CD4^+^CD25^+^Foxp3^+^ [29]. It has been shown that BM-MSCs secrete PGE_2_ in response to IFNγ stimulus [14]; because we detected the presence of IFNγ in all co-cultures, we evaluated the concentration of PGE_2_ in the conditioned medium of MSCs cultured in the absence and presence of activated T-cells. The concentration of PGE_2_ detected in the conditioned medium of MSCs cultured in the absence of activated T-cells was considered as the basal expression of this molecule. We observed that the basal secretion of PGE_2_ by DT-MSCs (DP-MSCs: 9.77 ± 2.79 ng/mL, G-MSCs: 9.71 ± 2.22 ng/mL and PDL-MSCs: 6.89 ± 1.55 ng/mL) was significantly higher (*p* < 0.05) than that secreted by BM-MSCs (0.34 ± 0.31 ng/mL) (Figure 5b). However, in the presence of activated T-cells, BM-MSCs significantly increased (*p* < 0.05) PGE_2_ secretion (11.55 ng/mL ± 1.29), but this value was similar to that observed in DT-MSCs under similar conditions (DP-MSCs: 12.39 ± 2.99 ng/mL, G-MSCs: 12.12 ± 1.49 ng/mL, and PDL-MSCs: 12.66 ± 2.44 ng/mL), (Figure 5b). These results indicate differences in the basal secretion of PGE_2_ between BM-MSCs and DT-MSCs; however, in the presence of activated T-cells, this secretion increases in BM-MSCs and reaches values similar those in DT-MSCs, which indicates that this molecule may be involved in inhibiting T-cell proliferation in co-cultures.

### 3.5. MSCs from Dental Tissues Induce a Similar Generation of T-cell Subsets Displaying a Regulatory Phenotype

**Figure 6:**
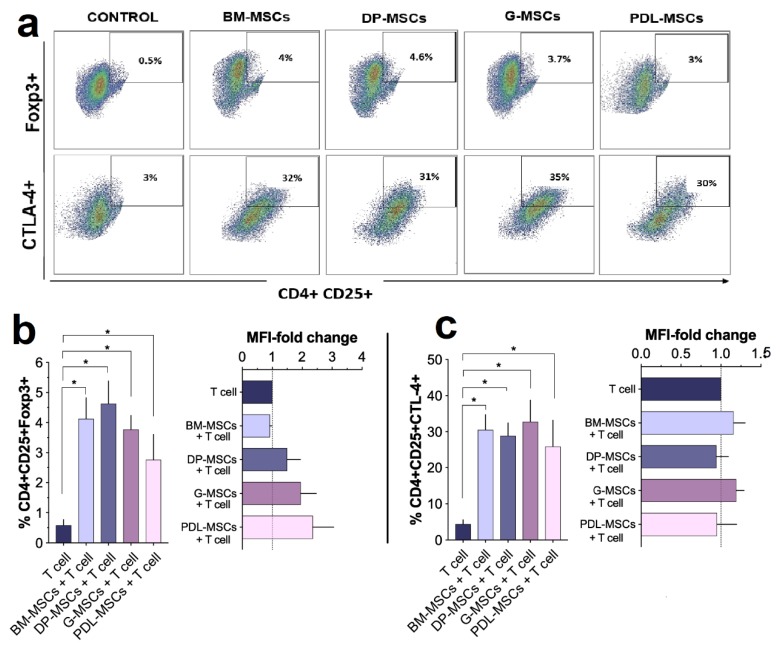
MSCs from dental tissues similar to those from BM induce the generation of T-cell subsets displaying a regulatory phenotype. CD3^+^ T-cells activated with anti-CD3/CD28 were co-cultured in the absence and presence of BM-MSCs or DP-MSCs or G-MSCs or PDL-MSCs at a 1:1 ratio of MSCs:T-cells (n = 5). The average percentage of Foxp3^+^ cells and CTLA-4^+^ cells, along with the fold increase in MFI of Foxp3^+^ or CTLA-4^+^ cells, were determined in the CD4^+^CD25^+^ population after four days of culture. The CD4^+^CD25^+^Foxp3^+^ and CD4^+^CD25^+^CTLA-4^+^ populations determined from cultures in the absence of MSCs were considered as the control. (**a**) Dot plots representative of one experiment. (**b**) Data are shown as the mean ± SEM of the percentage of CD4^+^CD25^+^Foxp3^+^ cells and the fold increase in Foxp3^+^ MFI in the CD4^+^CD25^+^Foxp3^+^ population. (**c**) Data are shown as the mean ± SEM of the percentage of CD4^+^CD25^+^CTLA-4^+^ cells and the fold increase in CTLA-4^+^ MFI in the CD4^+^CD25^+^CTLA-4^+^ population. * Significant difference at *p* < 0.05.

Several studies have shown that molecules, such as IL-10, IL-4, and PGE_2_, are involved in the induction of differentiation into regulatory T-cells [22,26,30]. Since we identified increased levels of these mediators in co-cultures with MSCs, which are related to the inhibition of CD4^+^ and CD8^+^ T-cell proliferation and an increased frequency of CD4^+^CTLA-4^+^ cells, we assessed whether these findings were related to the generation of regulatory CD4^+^CD25^+^CTLA-4^+^ and/or CD4^+^CD25^+^ Foxp3^+^ T-cells (Figure 6a–c). For this, T-cell populations were obtained from co-cultures, and the percentages and MFI of Foxp3^+^ cells and/or CTLA-4^+^ cells within the CD4^+^CD25^+^ fraction were determined. In lymphocytes activated in the absence of MSCs, the percentage of CD4^+^CD25^+^ T-cells positive for Foxp3 or CTLA-4 and the MFI of both molecules were determined and considered as basal values for T-cells with a regulatory phenotype (control). The results show that the presence of MSCs from all sources significantly increased (*p* < 0.05) the percentage of the CD4^+^CD25^+^Foxp3^+^ population (BM-MSCs: 4.13% ± 1.22; DP-MSCs: 4.64% ± 1.30; G-MSCs: 3.77% ± 0.81, and PDL-MSCs: 3.33% ± 1.25) compared with the control (0.54% ± 0.37) (Figure 6a,b). However, the presence of MSCs did not favor a significant increase in Foxp3 MFI in CD4^+^CD25^+^ T-cells compared with the control (Figure 6a,b).

Similar results were observed with the four sources of MSCs regarding the generation of CD4^+^CD25^+^CTLA-4^+^ regulatory cells because their presence significantly increased (*p* < 0.05) the percentage of this population (BM-MSCs: 32.30% ± 7.03; DP-MSCs: 31% ± 5.71, G-MSCs: 35.33% ± 10.87, and PDL-MSCs: 28.55% ± 7.3) compared with the control (3.23% ± 0.8); an increase in CTLA-4 MFI in CD4^+^CD25^+^ T-cells compared with the control was not detected (Figure 6c). These results indicate that DT-MSCs and BM-MSCs have a similar capacity to generate populations with regulatory phenotypes and that these populations may be involved in the decrease in T-cell proliferation.

## 4. Discussion

Currently, because of their immunoregulatory capacity, BM-MSCs have been used as a treatment for GVHD—a condition in which donor T-cells are the main effector cells participating in the immune response against host tissues [31]. BM-MSCs are the most used cells in clinical protocols; however, they have the disadvantage of being obtained by invasive procedures [9]. Notably, the presence of MSCs in dental tissues has been demonstrated [32,33], and the MSCs can be obtained through minimally invasive isolation techniques and even show an adequate proliferative capacity for expansion [34]. Different research teams have conducted in vitro studies with MSCs from different dental tissue sources and have reported immunosuppressive effects on those obtained from DP [35], G [15], and PDL [16]; however, the mechanisms by which they exert these functions have not been clearly established. To date, there is no in vitro study, under identical culture conditions, that compares the biological properties of MSCs from these sources and determines which MSC type shows the highest immunoregulatory capacity, what mechanisms are associated with it, and if they share immunoregulatory properties with BM-MSCs, to determine if they can be considered alternative sources of MSCs for clinical protocols.

In the present in vitro study, we showed that BM-MSC, DP-MSCs, G-MSCs, and PDL-MSCs met the criteria established by the ISCT [7] regarding the morphology, immunophenotype, and capacity for osteogenic, chondrogenic, and adipogenic differentiation [36,37]; however, we observed a decreased adipogenic differentiation potential in DP-MSCs compared to MSCs from other sources, given the detection of small vacuoles within the cytoplasm of cells with fibroblastoid morphology, coinciding with what we previously reported for cervix MSCs [28]. In fact, it has also been reported that some sources of MSCs, such as UCB and adipose tissue, have decreased or no adipogenic differentiation [13,38].

Previous studies have established immunoregulatory effects of BM-MSCs in vitro in co-culture systems with total PBMCs [39] or in mixed lymphocyte reactions [40]; in fact, this same system has been used with DP-MSCs [35], G-MSCs [15] and PDL-MSCs [16]. However, few studies have analyzed this effect on CD3^+^ T-cell-enriched populations [41,42], which is important in the context of GVHD because this effector population is the most important in this disease [31]. To evaluate this, we analyzed the immunosuppressive effect of DT-MSCs on one CD3^+^ T-cell-enriched population. We observed that DT-MSCs were able to decrease the proliferation of CD3^+^ T-cells activated with anti-CD3/CD28; similar results were previously obtained by our team with BM-MSCs [18]. When we examined if the CD4^+^ and CD8^+^ populations are equally affected by DT-MSCs; we found that all significantly reduce the proliferation of both. These in vitro results are consistent with previous research showing that DP-MSCs, G-MSCs, and PDL-MSCs have immunosuppressive effects on PBMCs activated with PHA [15,16,35]. G-MSCs are the most studied dental tissue cells, primarily for their effects on tissue regeneration in maxillofacial surgery [33], and have been shown to decrease T-cell proliferation in in vitro and in vivo models, in addition to increasing the population of Tregs in joints, spleen, and peripheral blood and decreasing the Th17 population and the production of proinflammatory cytokines [43]. Furthermore, DP-MSCs [35], G-MSCs [41], and PDL-MSCs [16] are able to decrease T-cell proliferation via transforming growth factor-b1 (TGF-β) in vitro [35] and by the induction of apoptosis via (FasL)/Fas in vivo [43] as well as in a collagen-induced arthritis (CIA) model [44]. Our results demonstrate for the first time, under identical culture conditions, that BM-MSCs, DP-MSCs, G-MSCs, and PDL-MSCs have the same potential to immunosuppress CD3^+^ T-cells activated with anti-CD3/CD28.

Previous in vitro studies in our laboratory have shown that BM-MSCs have immunoregulatory effects on T-cells through direct cell–cell contact between the two populations by the participation of surface molecules [18]; however, this aspect is unknown with DT-MSCs. Due to this, we evaluated some surface molecules involved in the mechanism of immune regulation in both DT-MSCs and CD3^+^ T-cells. In this sense, the expression of the adhesion molecules intracellular adhesion molecule (ICAM)-I and vascular cell adhesion molecule (VCAM)-I in MSCs favors the decrease in proliferation in splenocytes activated with anti-CD3 antibodies [45]; additionally, both adhesion molecules can induce the expression of cytotoxic T lymphocyte-associated antigen-4 (CTLA-4), which is related to the suppressive action and to the inhibition of T-cell proliferation [46,47]. We found that after four days of culture, the expression of CTLA-4 in T-cells of co-cultures with MSCs from all four sources significantly increased, coinciding with the decrease in T-cell proliferation; this suggests the participation of CTLA-4^+^ Tregs for this inhibition to occur. These observations are consistent with previous research, showing that BM-MSCs, UCB-MSCs, and PL-MSCs have immunosuppressive capacities by increasing CTLA-4 in co-cultures with CD3^+^ [18,48].

Likewise, the decrease in T-cell proliferation in co-cultures with MSCs may be related to the expression of PD-L1, which is involved in immunosuppression mediated by MSCs through cell contact-dependent mechanisms [20]; in fact, the PD-L1/PD-1 signaling pathway is considered an immune checkpoint in therapy for inflammation and cancer [49]. Currently, only PD-L1 has been reported in DP-MSCs [50]; this could be because dental tissues are exposed to constant immunological challenge, and thus, tolerance mechanisms increase, as in periodontitis—the most common oral pathology caused by bacterial infections—which can be resolved by PD-L1 negatively regulating inflammation and maintaining periodontal tissue under constant immunological tolerance [17]. PD-L1 represses the maturation of Th17 and promotes CD4^+^CD25hi Foxp3^+^ Treg differentiation [51]. This is one of the main mechanisms by which MSCs are considered non-immunogenic and establishes the reason for therapy in immune-mediated diseases such as GVHD, autoimmune encephalomyelitis, multiple sclerosis, type 1 diabetes, rheumatoid arthritis, systemic lupus erythematosus, Crohn’s disease, and cirrhosis, among others [52,53]. Our results show, under the co-culture conditions studied, that MSCs from the four cell sources significantly increased PD-L1 expression levels compared with their basal state. Similar results have been observed in BM-MSCs, UCB-MSCs and PL-MSCs [18] and Wharton’s jelly [54]. Notably, DP-MSCs have constitutively higher levels of PD-L1 compared with MSCs from the other sources and an even greater expression of PD-L1 after co-culture with activated T-cells, which is an interesting aspect which we are studying and could be related to a different genetic signature from the other MSCs evaluated. Thus, in the present in vitro study, it was observed for the first time, under the same co-culture conditions, that BM-MSC, DP-MSCs, G-MSCs and PDL-MSCs have the potential to increase the surface expression of PD-L1 and of CTLA-4 on CD3^+^ T-cells activated with anti-CD3/CD28; both mechanisms participate in the decrease in proliferation observed in the latter.

IFNγ and TNFα have been shown to stimulate the synthesis of molecules such as IDO, PGE2, and PD-L1 in MSCs [20], thus favoring the immunosuppressive effect of these cells [55]. Due to this, we analyzed the expression of both cytokines in the co-culture supernatants of each source of MSCs. Previous studies suggest that IFNγ and TNFα stimulate IDO secretion in placental MSCs [56] and in those MSCs from human exfoliated deciduous teeth (SHED) (SHED) [57]; this enzyme depletes tryptophan, catalyzing its degradation in the kynurenine (KYN) pathway. Both the reduction in local tryptophan concentration and the generation of KYN metabolites contribute to the immunoregulatory effects of IDO. IDO expression in antigen-presenting cells (APCs) has been suggested to induce the secretion of IL-10 by Tregs [58], promoting the inhibition of proliferation of CD4^+^ and CD8^+^ T-cells [57,58]. Certain studies suggest that an inflammatory stimulus, through IFNγ, TNFα, CoX-2, and IDO, is a prerequisite for MSCs to have immunosuppressive properties. Our results showed the presence of IFNγ and TNFα in co-cultures. As described in other studies with BM, UCB, PL, adipose tissue (AT) and Wharton’s jelly [20,59], even despite low IFNγ and TNFα production, it was found that the concentrations detected are sufficient to stimulate the immunosuppressive capacity in MSCs and, thus, inhibit T-cell proliferation.

Similarly, the decrease in T-cell proliferation observed in co-cultures could be related to the presence of immunosuppressive cytokines. It has been observed that T-cell activation in the presence of BM-MSCs induces the secretion of Th2 anti-inflammatory cytokines (IL-4 and IL-10). Moreover, TNFα in combination with IFNγ increases the immunosuppression mediated by MSCs by promoting the secretion of other immunosuppressive molecules such as PGE_2_ [14,19]. Therefore, because of the observed pattern of production of IFNγ and TNFα in co-culture supernatants, we also analyzed the presence of IL-10, IL-4, and PGE_2_. Our results showed a significant increase in the expression of IL-4 in BM-MSCs compared with DT-MSCs, suggesting that DT-MSCs may not have the ability to generate a Th2-type response. The increase in IL-4 has been reported to promote Treg differentiation [60]. On the other hand—and contrary to the detected levels of IL-4—we observed a significant increase in the production of IL-10 in co-cultures of DT-MSCs compared with BM-MSCs; these observations are consistent with previous research, in which it was determined that AT-MSCs have immunosuppressive capabilities by increasing IL-10 in vitro and in vivo in a CIA model [61].

Studies conducted in co-cultures of minipig BM-MSCs with MLR also demonstrated that PGE_2_ can stimulate the secretion of IL-10 and IFNγ, favoring Treg differentiation [25]. Using antibodies, IL-10 has been determined to be produced by MSCs and is important for exerting immunosuppressive effects on T-cells and inducing Treg differentiation [21]. Similarly, it has been shown that PGE_2_ stimulates the secretion of IL-10 and IL-4, favoring a decrease in T-cell proliferation and induction to CD4^+^CD25^+^Foxp3^+^ lymphocytes [29,61]. The immunomodulatory effect of MSCs on allogeneic responses can been explained in part by cyclooxygenase-2 (COX-2) derived from PGE_2_. The biosynthesis of PGE_2_ by MSCs is not only constitutive but also induced under inflammatory stimuli [4,18]. Our in vitro results showed that DT-MSCs constitutively expressed significantly higher concentrations of PGE_2_ than BM-MSCs; however, in co-culture with activated T-cells, we observed similar concentrations in the four sources studied. Dental tissues have an immune profile associated with mucous membranes, unlike BM, and this could be related to the difference in basal production of PGE_2_ by DT-MSCs compared with BM-MSCs; in fact, it has been described that PGE_2_ is necessary for G-MSCs to perform immunosuppressive functions through the prostaglandin E_2_–EP_3_ signaling pathway [61]. Due to the variability in the expression pattern of soluble molecules identified in the co-culture supernatants of MSCs with CD3^+^ activated with anti-CD3/CD28, it can be hypothesized that BM-MSCs and DT-MSCs, under identical culture conditions, exert their immunosuppressive effect by different mechanisms, which could be determined by their histological origin.

Because we observed high concentrations of IL-10 and PGE_2_ in co-cultures, in addition to IFNγ secretion, and because it has been shown that these molecules favor the generation of Tregs [60], we decided to detect the presence of Tregs in our co-cultures. Tregs are characterized by their suppressive and anergic properties. The two most relevant classes of CD4^+^ Tregs are the CD4^+^CD25^+^ Foxp3^+^ and CD4^+^CD25^+^CTLA-4^+^ lineages [25]; therefore, we decided to identify them. Our in vitro results indicate a significant increase in both populations compared with the levels detected in the non-activated T-cell subset. These results coincide with those obtained for DP-MSCs in co-cultures with CD4^+^ T-cells where an increase in the CD4^+^ CD25^+^ Foxp3^+^ population is observed [62]. This is the first study showing that MSCs from DP, G, and PDL similarly induce the generation of Tregs in vitro under these co-culture conditions. Periodontal tissue has Tr1-like T-cells—a type of Treg recently described patrolling peripheral tissue [22]. Similarly, it has been reported that the presence of PDL-MSCs and G-MSCs in co-cultures with activated PBMCs increases the CD4^+^ CD25^+^ Foxp3^+^ population and suppresses T-cell effector cells, as with Th1/Th2/Th17 populations [63]. Paradoxically, it has been reported that G-MSCs in vitro are not capable of generating Tregs; however, after their application in an acute GVHD model, high levels of Foxp3 expression were detected, along with a reduction in the levels of proinflammatory cytokines IL-17 and IFNγ and an increase in IL-2 levels, the role of which is important for the differentiation of induced regulatory T-cells (iTregs), supporting the suppressor function of CD4 iTregs under inflammatory conditions [64]. Recent studies have shown that the expression of CD39 on CD4^+^ CD25^+^ Foxp3^+^ Tregs catalyzes the sequential generation of adenosine by degradation of extracellular ATP/ADP by CD39 and conversion of 5’-AMP by CD73 [65,66]. These events lead to a marked reduction in T-cell proliferation and secretion of proinflammatory cytokines. Interestingly, it has been demonstrated that G-MSCs not only express CD39 but also increase the frequency of CD39^+^Foxp3^+^ Tregs, which supports the generation of adenosine and promotes immune suppression of effector T-cells in vitro and in an acute GVHD model [64]. Currently, in our laboratory, we are studying the generation of different Treg subsets by MSCs from the different sources of dental tissues used in this study.

In summary, our study shows differences in the production of cytokines and surface molecules that may participate in T-cell immunosuppression by DT-MSCs. However, interestingly, MSCs from the dental sources evaluated presented the same capacity to reduce the proliferation of CD4+ and CD8+ populations, which indicates that any of the three sources of DT-MSCs would be appropriate for in vivo immunoregulatory applications against T-cells, which is important because they are effector cells in immunological diseases such as graft-versus-host disease. Nevertheless, it is necessary to evaluate the immunosuppressive capacity of DT-MSCs against other cells of the immune system to determine if the functional differences we have observed are required for the suppression of the immune activity of a particular cell.

## 5. Conclusions

This is the first study demonstrating, under the same culture conditions, that MSCs from DP, G, and PDL have the same in vitro capacity to decrease the proliferation of activated CD3^+^ T-cell-enriched populations and that this capacity is similar to that observed with BM-MSCs. Additionally, we demonstrated differences in the production of cytokines and surface and secreted molecules that may participate in T-cell immunosuppression in co-cultures in the presence of DT-MSCs compared with BM-MSCs; however, it is important to mention that the four sources studied had the same capacity to induce the generation of CD4^+^CD25^+^Foxp3^+^ and CD4^+^CD25^+^CTLA-4^+^ T-cell subsets displaying a regulatory phenotype. MSCs have become one of the most attractive cell types for cell therapy due to their immunosuppressive properties. At present, several clinical trials involving the use of MSCs for GVHD and autoimmune diseases are in progress. However, because dental tissues are small sources of MSCs, it is necessary to use explant cultures to obtain individual MSCs, which can be cultivated afterwards in successive passages to expand the cells to reach the number needed for clinical applications (1–5 × 10^6^/kg) [67]. Different groups have been able to expand MSCs on a clinical scale and in sufficient numbers for their use in clinical trials [68,69], which can be applied in the case of MSCs from dental tissues. In light of our in vitro results, we propose that, in addition to BM-MSCs, DT-MSCs might be reliable candidates for future therapeutic applications for the treatment of immunological diseases, such as GVHD, graft rejection, or autoimmune diseases. Finally, although it is important to determine the in vitro immunoregulatory potential of DT-MSCs, it is necessary to evaluate these capacities in animal models to analyze the decrease of proinflammatory cytokine production by MSC transplantation. These experiments are being planned for future studies. 

## Supplementary Materials

The supplementary materials are available online at https://www.mdpi.com/2073-4409/8/12/1491/s1.
