# Supplementary material for: Mesenchymal Stem/Stromal Cells Derived from Dental Tissues: A Comparative In Vitro Evaluation of Their Immunoregulatory Properties Against T cells
Source: Cells. 2019 Nov 22;8(12):1491. doi: 10.3390/cells8121491 (PMC6953107; doi:10.3390/cells8121491)
Supplement: Supplementary file 1 [file cells-08-01491-s001.pdf]

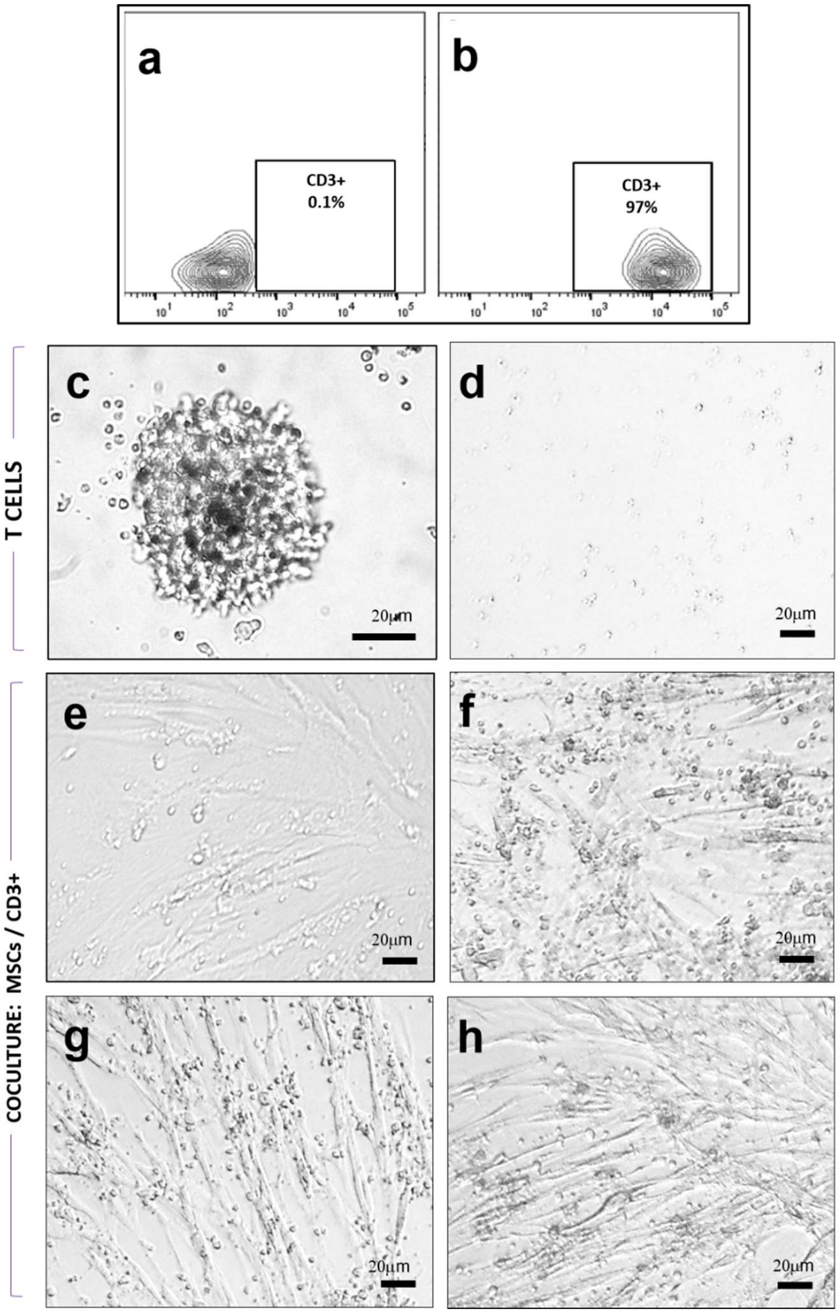

Figure S1. ***The purity of CD3+ T-cells and cocultures of MSCs/CD3+ T-cells.*** **(a)** Representative dot plot of the percentage of CD3+ positive cells in total PBMCs. **(b)** Representative dot plot of the purity (≥97%) of the obtained CD3+ T-cell suspensions by separation with human CD3 MicroBeads and MACS MS columns (Miltenyi Biotec, Bergisch Gladbach, Germany). Representative cocultures and controls. **(c)** $CD3^+$ T-cells activated with anti-CD3/CD28 in the absence of MSCs (control). **(d)** $CD3^+$ T-cells not activated in the absence of MSCs (control). **(e)** BM-MSCs/activated T-cells coculture. **(f)** DP-MSCs/activated T-cells coculture. **(g)** G-MSCs/activated T-cells coculture. **(h)** PDL-MSCs/activated T-cells coculture.

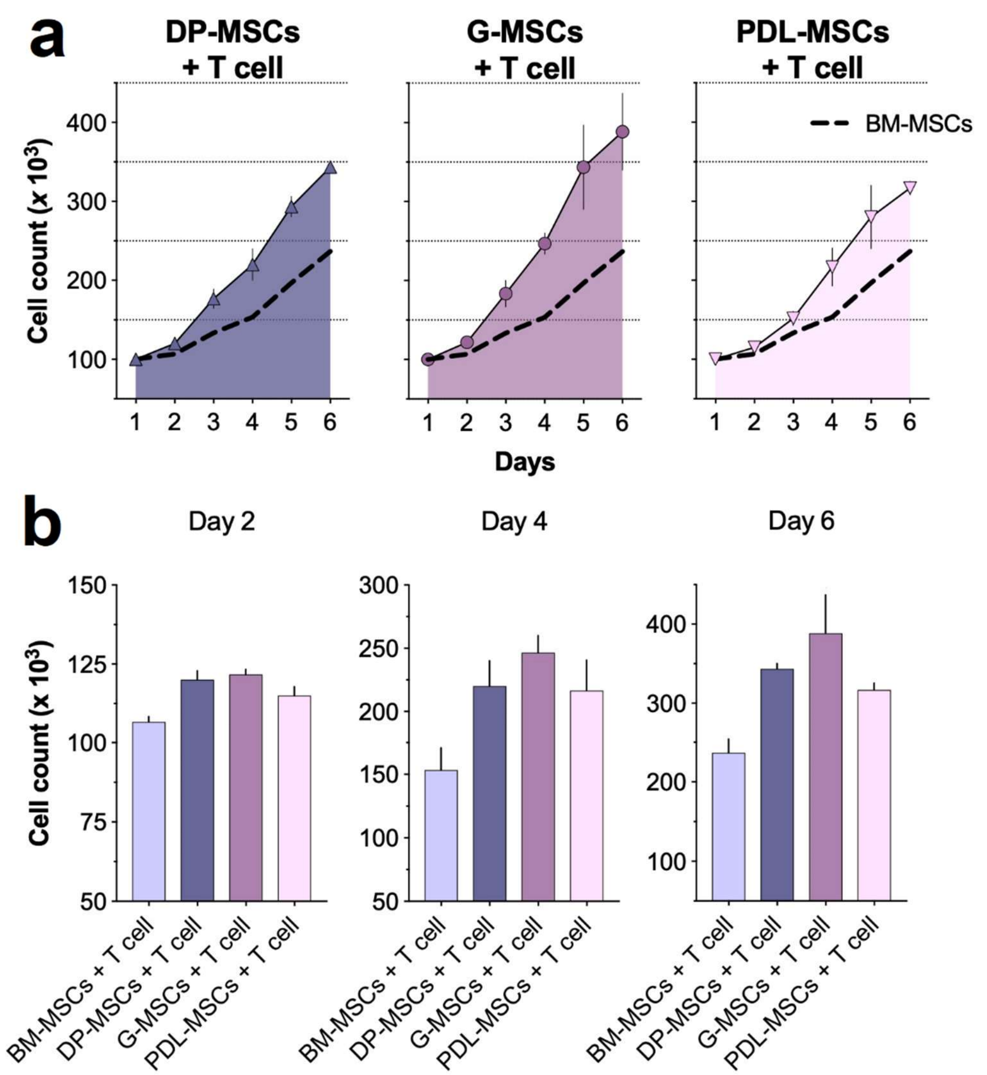

Figure 2. ***Similar proliferation kinetics of MSCs from dental tissues and bone marrow.*** Culture were established from DT-MSCs and BM-MSCs and sustained from day 1 to day 6. Cells were cultured in 6-well tissue culture plates at an initial concentration of 1 x 10^3 cells/well. MSCs were harvested daily from the wells and counted by using a hemocytometer. **(a)** Symbols represent mean ± SEM from five separate experiments (n=5), from each indicated source. **(b)** Comparison between the different sources on days 2, 4 and 6. Data are shown as the mean ± SEM of cell number.
